# Fluorescent Protein Expression as a Proxy for Bacterial Fitness in a High-Throughput Assay

**DOI:** 10.1128/AEM.00982-21

**Published:** 2021-08-26

**Authors:** Rudolf O. Schlechter, Evan J. Kear, Daniela M. Remus, Mitja N. P. Remus-Emsermann

**Affiliations:** a School of Biological Sciences, University of Canterbury, Christchurch, New Zealand; b Biomolecular Interaction Centre, University of Canterbury, Christchurch, New Zealand; c Bioprotection Research Core, University of Canterbury, Christchurch, New Zealand; d Protein Science and Engineering, Callaghan Innovation, School of Biological Sciences, University of Canterbury, Christchurch, New Zealand; e Institute of Biology, Freie Universität Berlin, Berlin, Germany; Chinese Academy of Sciences

**Keywords:** competition, optical density, mScarlet, sYFP2

## Abstract

Bacterial growth is classically assessed by measuring the increases in optical density of pure cultures in shaken liquid media. Measuring growth using optical density has severe limitations when studying multistrain interactions, as it is not possible to measure the growth of individual strains within mixed cultures. Here, we demonstrated that constitutively expressed fluorescent proteins can be used to track the growth of individual strains in different liquid media. Fluorescence measurements were highly correlated with optical density measurements and cell counts. This allowed us to assess bacterial growth not only in pure cultures but also in mixed bacterial cultures and determine the impact of a competitor on a focal strain, thereby assessing relative fitness. Furthermore, we were able to track the growth of two different strains simultaneously by using fluorescent proteins with differential excitation and emission wavelengths. Bacterial densities measured by fluorescence yielded more consistent data between technical replicates than optical density measurements. Our setup employs fluorescence microplate readers that allow high throughput and replication.

**IMPORTANCE** We expand on an important limitation of the concept of measuring bacterial growth, which is classically limited to one strain at a time. By adopting our approach, it is possible to measure the growth of several bacterial strains simultaneously with high temporal resolution and in a high-throughput manner. This is important to investigate bacterial interactions, such as competition and facilitation.

## INTRODUCTION

Measurement of bacterial growth in liquid culture is a central paradigm for microbiology. Growth is specific for every bacterial strain, and growth rates are determined as part of every characterization of novel bacterial species or to describe the impact of mutations on bacterial fitness (1). Classically, growth is assessed by determining changes in the turbidity and absorbance of liquid cultures using standards like the McFarland standard (2) or by taking turbidity measurements with a spectrophotometer (3). These methods can be used to determine growth rates and predict the cell numbers or maximal optical densities (ODs) of individual strains or whole communities in liquid medium. However, they suffer from limitations, as spectrophotometers require bacterial cells to be well mixed without any formed clumps that would increase light scattering or cause heterogeneous turbidity (4). Thus, measuring the growth of bacterial species that form aggregates in liquid media can be problematic. Furthermore, it is not possible to determine the growth of more than one bacterial strain in the same culture, since only their combined optical density can be assessed. As an alternative to optical density, fluorescent dyes have been used to determine cell densities, e.g., the addition of the DNA intercalating dye acridine orange has been used to determine cell densities (5). This is possible by fluorometric analysis of liquid samples. In addition to chemical dyes, fluorescent proteins can be detected in fluorometric analysis.

The green fluorescent protein (GFP) has been used in molecular microbiological experiments since the nineties (6, 7). Since then, many additional fluorescent proteins have been discovered or developed (e.g., see references 8 and 9). Many of these new proteins have dramatically different properties, such as improved brightness and photostability but also different spectral excitation and emission properties (10–12). Using GFP as a proof of concept, it has been demonstrated that constitutively expressed fluorescent proteins can be used to predict the growth and CFU count of Pseudomonas aeruginosa (13). This encouraged us to pursue a similar approach to predict the growth of bacteria in different liquid media and in coculture with other differentially tagged bacteria.

In this work, we demonstrated the use of different constitutively expressed fluorescence proteins as a means to measure bacterial growth. Furthermore, we used proteins with differential excitation and emission spectra (14) to allow the measurement of several populations at the same time. We performed our experiments in 96-well microtiter plates and microtiter plate readers, which allowed time series monitoring of fluorescence signals and cell growth under different growth conditions with a high degree of reproducibility. By combining these measurements with a series of controls, we demonstrated that our system allowed us to determine the strength of interaction and relative fitness of bacterial strains in competition.

## RESULTS

### Growth of Pe::red in nutrient broth.

**FIG 1:**
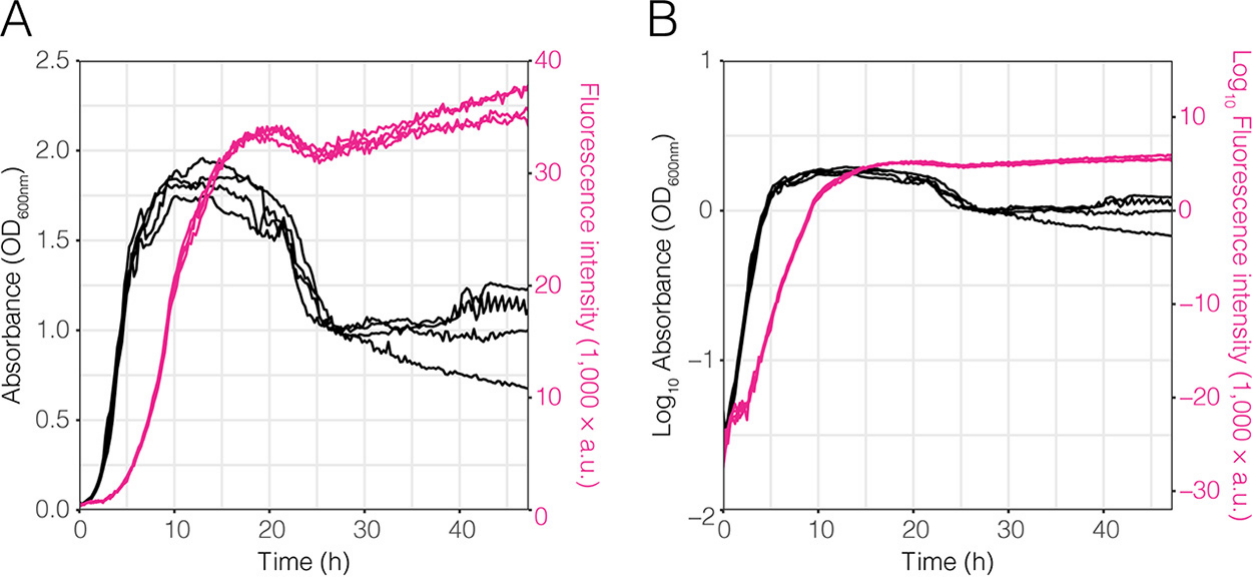
Pe::red growth in nutrient broth. Growth of Pe::red in nutrient broth in a 96-well microtiter plate was tracked using optical density (black) and fluorescence intensity (magenta). Optical density was measured as the absorbance of the culture at 600 nm (OD_600_), and red fluorescence was measured at 613 to 626 nm (A.U., arbitrary units). Data are expressed in linear (A) and logarithmic (B) scale.

To highlight the advantage of fluorescence intensity measurements over optical density measurements, we followed the growth of Pantoea eucalypti strain 299R labeled with a constitutively expressed red fluorescent protein (strain Pe::red) in five replicated cultures using optical density and fluorescence intensity in nutrient broth (NB) (Fig. 1A). The optical density increased exponentially for ∼5 h (Fig. 1B) to an OD of 1.2. Afterwards, the culture entered a stationary phase for roughly 20 h, followed by a rapid decline in optical density. The corresponding fluorescence increased exponentially for ∼10 h (Fig. 1B) to a fluorescence intensity of 20,000 arbitrary units (A.U.). Afterwards, the rate with which fluorescence intensity increased was slightly reduced, indicating that cell growth decreased. After 20 h, the fluorescence intensity peaked at about 35,000 A.U. After a small reduction of fluorescence, suggesting cell lysis, the fluorescence increased again until the end of the experiment at 48 h. Growth curves obtained through optical density deviated even though the cultures were seeded from the same original culture and were diluted to the same initial optical density. The deviations were as large as an OD at 600 nm (OD_600_) of 0.25, which is more than 10% between the individual cultures. In contrast, the fluorescence measurements of the individual cultures exhibited much smaller deviations, showing that measuring fluorescence yielded more consistent results.

Furthermore, after 20 h of growth in 96-well plates, the optical density of Pe::red decreased, while red fluorescence steadily increased over time (Fig. 1A). To determine whether cell lysis or flock formation was associated with this growth pattern, independent cultures of Pe::red grown in NB were sampled over time and observed under the microscope for qualitative analysis. At early time points, that is, from 0 to 4 h, most cells were planktonic and emitted fluorescence (Fig. S1). However, we observed both cell lysis and flock formation at 24 h, as a proportion of cells showed loss of fluorescence and morphological changes indicative of cell lysis in the phase-contrast images (Fig. S1 in the supplemental material), while other cell subpopulations aggregated (Fig. S2).

### Growth of Pe::red in diluted NB.

**FIG 2:**
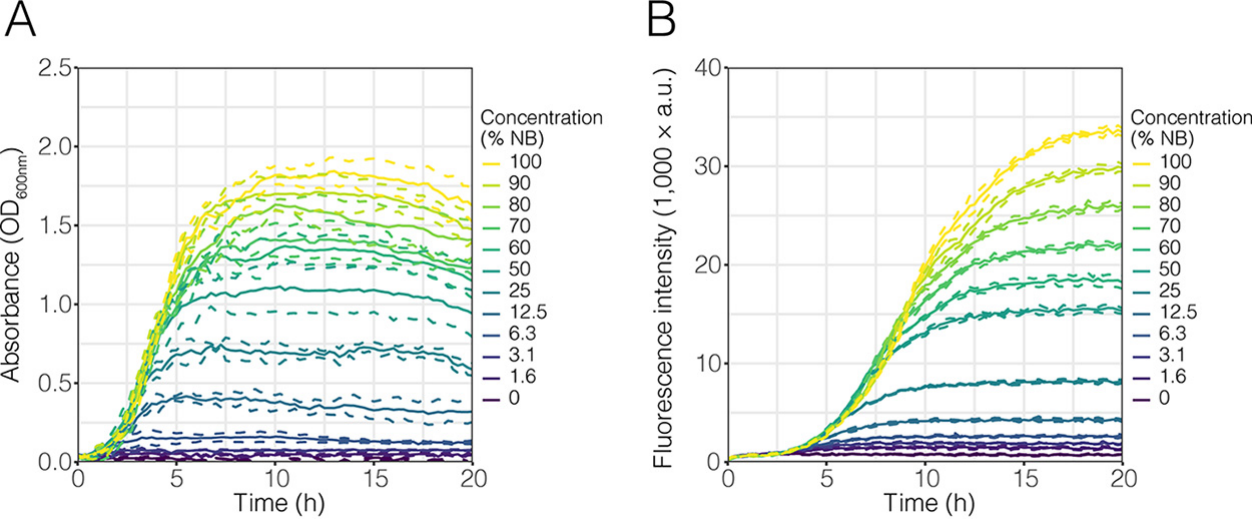
Growth of Pe::red on different concentrations of nutrient broth. Pe::red was inoculated into 96-well microtiter plates containing increasing concentrations of nutrient broth (NB), ranging from 0% to 100% (vol/vol) strength (see keys). Growth was measured by optical density at 600 nm (OD_600_) (A) and fluorescence intensity emission (A.U., arbitrary units) (B) as proxies. In both cases, continuous lines represent the mean values from four biological replicates and dashed lines represent the standard deviations (SD).

After inoculation into different concentrations of NB, Pe::red grew to different final optical densities, while the initial lag phase and growth were similar (Fig. 2A). As expected, the final optical density was directly dependent on the concentration of the NB medium. Depending on the available resources, the different cultures reached their stationary phases at different times and densities. Pe::red grown on 6.75% strength NB reached stationary phase after ∼2.5 h, while cultures growing on 12.5, 25, and 50% strength NB reached stationary phase after 5 h. Cultures growing on higher concentrations of NB reached stationary phase between 7.5 and 10 h. Worthy of note are the large standard deviations and irregular curves, especially after the cultures entered their respective stationary phases. This was more pronounced at higher optical densities. After 15 h, some of the cultures exhibited small declines in optical density.

For fluorescence intensity measurements, the same cultures exhibited increases in fluorescence over time in increasing concentrations of NB that were similar to the increases of the optical densities (Fig. 2B). As expected, the increase in fluorescence lagged the increase in optical density, likely due to the fluorescent protein maturation rate. The overall ranking of maximal fluorescence levels was the same as for the optical density measurements. The variability between the replicates was minimal in comparison to the variability of the optical density data. The fluorescence data did not exhibit a decline compared to the decline in the optical density data and remained stable until the end of the experiment after 20 h.

### Correlating bacterial growth with optical density and fluorescence intensity.

Next, we tested whether constitutively expressed fluorescence signals could be used as a proxy for growth. To that end, we used different measures of the optical density and fluorescence data extracted from Fig. 2, including the area under the curve (AUC), the maximal value, and the final value for each curve. We fitted these data into simple linear regression models of fluorescence and optical density to determine the parameters that gave the best fit by comparing the adjusted *R*^2^ and Pearson’s correlations (Table 1). Generally, we observed that every linear regression fitted the data with an *R*^2^ of >0.95. The AUC of fluorescence data was the best predictor for every parameter of optical density used (i.e., AUC, maximum value, and final value), resulting in the best fits (*R*^2^ = 0.98) and highest Pearson’s correlation values (0.99) (Table 1).

**TABLE 1:** Parameters derived from linear regression models and correlation between the fluorescence and absorbance measurements presented in Fig. 2

Response variable^*a*^	Explanatory variable^*a*^	Adjusted *R*^2^	Pearson’s correlation
AUC OD	AUC RFU	0.98	0.99
AUC OD	Max RFU	0.95	0.98
AUC OD	Final RFU	0.95	0.97
Max OD	AUC RFU	0.98	0.99
Max OD	Max RFU	0.95	0.98
Max OD	Final RFU	0.95	0.98
Final OD	AUC RFU	0.98	0.99
Final OD	Max RFU	0.96	0.98
Final OD	Final RFU	0.96	0.98

a OD, optical density; RFU, relative fluorescence units; AUC, area under the curve; Max, maximum value of data set; Final, final value of data set.

**FIG 3:**
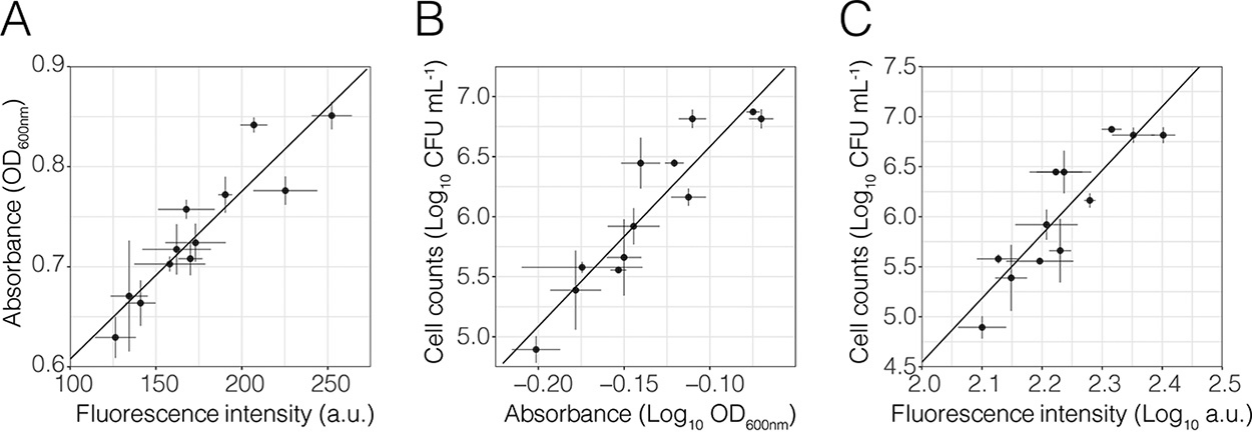
Relationships between fluorescence, absorbance, and cell counts. Fluorescently tagged Pe::red was grown in shaken conical flasks. Samples were taken after 0, 0.75, 1.50, 2.50, 3.25, and 4 h postinoculation. Each point represents the average value for three technical replicates for each time point and each biological replicate (*n* = 2), with error bars representing SD. Lines depict simple linear regression models. (A) Fluorescence versus absorbance (linear regression, *P* < 0.05, *R*^2^ = 0.84, Pearson’s correlation = 0.92). (B) Absorbance versus cell count (linear regression, *P* < 0.05, *R*^2^ = 0.84, Pearson’s correlation = 0.93). (C) Fluorescence versus cell count (linear regression, *P* < 0.05, *R*^2^ = 0.78, Pearson’s correlation = 0.89). (B and C) Cell counts were determined as CFU ml^−1^, and data are represented in logarithmic scale (log_10_). A.U., arbitrary units; OD_600_, optical density at 600 nm.

In addition, we compared the optical density, fluorescence, and colony counts of two independent Pe::red NB cultures that were grown in Erlenmeyer flasks and sampled over time. To test the changes in the levels of fluorescence intensity in Pe::red over time, the fluorescence emissions of individual Pe::red cells were measured and analyzed at different time points. We observed that from 0 to 4 h, single cells exhibited a statistically significant, albeit small, increase of fluorescence (Fig. S3). However, the low coefficient estimates derived from generalized linear models for each replicate, together with the low goodness-of-fit for each model (replicate 1, intercept = 940.85 A.U., coefficient estimate = 0.18, adjusted *R*^2^ = 0.0079; replicate 2, intercept = 532.75 A.U., coefficient estimate = 3.25, adjusted *R*^2^ = 0.13) suggested that growth time had only a small effect on single-cell fluorescence intensity, where about 10% of the data could be explained by these models. The results indicate that the fluorescence of individual cells increased at very low rates during early growth phases and that the increase in fluorescence in a population could be associated with an increase in the population density instead of an increase in the protein maturation rate. A strong correlation between fluorescence and optical density was observed between Pe::red cultures growing in NB sampled at different time points (Table 2, Fig. 3A). Similarly, both optical density and fluorescence intensity also correlated with bacterial counts, measured as CFU ml^−1^ (Fig. 3B and C, respectively).

**TABLE 2:** Parameters derived from linear regression models and correlation between the fluorescence and absorbance measurements presented in Fig. 3

Response variable	Explanatory variable	Adjusted *R*^2^	Pearson’s correlation
Fluorescence intensity	Optical density	0.84	0.92
Bacterial counts^*a*^	Fluorescence intensity^*a*^	0.78	0.89
Bacterial counts^*a*^	Optical density^*a*^	0.84	0.93

a Data were transformed to logarithmic scale (log_10_).

Finally, we evaluated the correlation between fluorescence and optical density in strains other than Pe::red and in different growth media. To that end, fluorescently tagged strains, listed in Table 3, were incubated in minimal medium (MM) supplemented with 31 different carbon sources, and both fluorescence emission and final OD_600_ were measured. We selected a wide range of phylogenetically different strains (from *Proteobacteria* and *Actinobacteria*) that constitutively express a red or green fluorescent protein. In general, we observed a statistically significant and positive correlation between the AUCs of the fluorescence intensity curves of bacterial strains and the final OD_600_ of each liquid culture (Fig. S4, generalized linear mixed model [GLMM], *P* < 0.05, pseudo-*R*^2^ = 0.80, Pearson’s correlation = 0.72).

**TABLE 3:** Bacterial strains used in this work

Strain	Short name; features^*a*^	Reference or source
Pantoea eucalypti 299R	PeWT	36
Pantoea eucalypti 299R::Tn*7*-mre145	Pe::red; red fluorescent, Gm^r^	14
*Sphingomonas* sp. Fr1	SpWT	37
*Sphingomonas* sp. Fr1::Tn*5*-mre143	Sp::yellow; yellow fluorescent, Gm^r^	This study
*Arthrobacter* sp. Leaf145::eGFP	Ar::green; green fluorescent, Cm^r^	This study
*Methylobacterium* sp. Leaf85::Tn*5*-mre145	Me85::red; red fluorescent, Gm^r^	This study
*Methylobacterium* sp. Leaf92::Tn*5*-mre145	Me92::red; red fluorescent, Gm^r^	14
Methylobacterium radiotolerans 0-1::Tn*5*-mre145	Mr::red; red fluorescent, Gm^r^	14
Sphingomonas phyllosphaerae FA2::Tn*5*-mre145	Sp2::red; red fluorescent, Gm^r^	This study

a Gm^r^, gentamicin resistant; Cm^r^, chloramphenicol resistant.

Consequently, data derived from the fluorescence signals allowed the tracking of growth in liquid cultures, showing that fluorescence can be used as an alternative to OD.

### Growth of Pe::red and Sp::yellow in the presence of different competitors.

**FIG 4:**
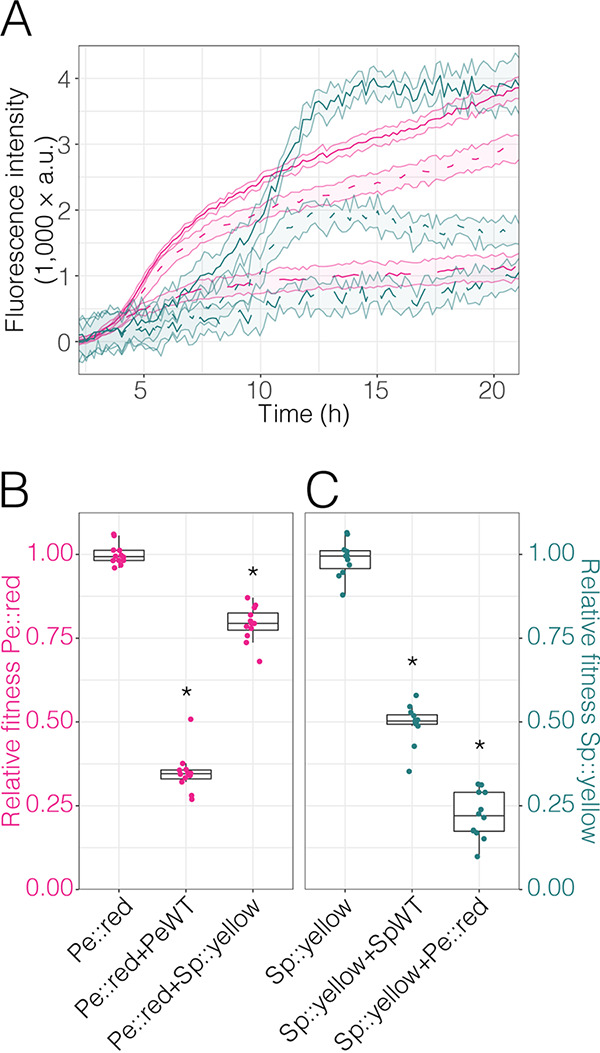
Competition assay in rich medium. The fluorescently tagged bacterial strains Pe::red and Sp::yellow were competed in nutrient broth, and their growth rates were estimated using fluorescence intensities. The data were background subtracted to normalize against the autofluorescence of the medium. (A) Fluorescence intensities over time for each strain under different conditions. Magenta, red fluorescence emitted by Pe::red; dark green, yellow fluorescence emitted by Sp::yellow. Solid lines represent monocultures of Pe::red or Sp::yellow. Broken lines represent each culture of Pe::red or Sp::yellow in competition with the respective wild-type PeWT or SpWT strains. Dotted lines represent each culture of Pe::red and Sp::yellow in coculture. Thin broken lines represent the standard deviations from the means. (B) Relative levels of fitness of Pe::red in monoculture, coculture with PeWT, and coculture with Sp::yellow. Fitness was determined as the AUC derived from red fluorescence relative to that of Pe::red in monoculture. (C) Relative levels of fitness of Sp::yellow in monoculture, coculture with SpWT, and coculture with Pe::red. Relative fitness of Sp::yellow was determined as the AUC derived from yellow fluorescence relative to that of Sp::yellow in monoculture. (B and C) Groups were compared using one-way ANOVA. *, *P* < 0.05 compared to the results for the monoculture (Bonferroni post hoc test).

To determine the impact of different competitors on the growth of Pe::red and Sp::yellow (*Sphingomonas* sp. strain Fr1 tagged with a constitutively expressed yellow fluorescent protein [YFP]), we inoculated mixed cultures into 96-well plates and covered the plates with a gas-permeable foil to avoid evaporation during long-term incubation. Each fluorescently tagged strain was mixed with its respective wild-type (WT) conspecific strain (Pe::red versus PeWT and Sp::yellow versus SpWT), and a mixture combining both fluorescently tagged strains (Pe::red versus Sp::yellow) was also used, allowing us to track the growth of each strain in parallel (Fig. 4A). As controls, the fluorescent strains were grown in monoculture. Their respective fluorescence levels over time were determined every 10 min. As expected, when grown without a competitor, Pe::red and Sp::yellow both reached the highest fluorescence intensity and, hence, the highest cell density (Fig. 4A, solid lines). When grown against the nearly isogenic wild-type strains, the fluorescence did not reach the same levels, depicting the impact of the competitor on the focal strain. We used the AUCs of the growth curves as proxies for bacterial abundance and to determine bacterial population sizes relative to those of their respective fluorescent monocultures. The relative AUC of the competition of Pe::red versus PeWT was on average 0.35 when normalized by the AUC of the Pe::red monoculture (Fig. 4B). Since conspecifics were expected to show no fitness differences when competing against each other, we evaluated the fitness of independent Tn*7*-insertion Pe::red mutant strains. The results from this experiment showed that the relative levels of fitness of Pe::red in competition with PeWT were consistent among strains and were usually 0.5 (Fig. S5). The relative AUC of Sp::yellow versus SpWT normalized by the AUC of the Sp::yellow monoculture was 0.49, and hence, Sp::yellow grew to almost exactly half the population density of the monoculture (Fig. 4C). When competing Pe::red and Sp::yellow, the relative AUCs of Pe::red and Sp::yellow were 0.79 and 0.22, respectively, when normalized by their respective monocultures (Fig. 4B and C). Hence, Pe::red had a competitive advantage over Sp::yellow and Sp::yellow was strongly impacted by the presence of Pe::red. Additionally, this competition assay was evaluated in MM supplemented with 0.2% (wt/vol) succinate, a resource that both strains can grow on. We observed a different pattern in which Pe::red in competition with Sp::yellow did not differ from Pe::red grown as monoculture (Fig. S6A), while Sp::yellow was affected by the presence of Pe::red (Fig. S6B). These results highlighted the applicability of this approach to different experimental setups to study the dynamics of bacterial interactions simultaneously and to address hypotheses regarding species interactions under defined growth conditions.

## DISCUSSION

We have designed an experimental platform that allows tracking of bacterial growth in 96-well plates using fluorescence measurement as an alternative to optical density. This allowed us to track distinct populations in parallel, and we provide an example of a mixed culture of two populations. Fluorescence measurements in plate readers also have the advantage of being less sensitive than optical density to cell aggregation, as several measurements of the same culture are taken and averaged, thereby integrating large areas of the sample and accounting for aggregates and less dense areas of the sample. In conjunction with controls, this approach allows for the measurement of competition between strains in a high-throughput manner. With optimized detector systems like filters or monochromators and well-chosen fluorescent proteins, it would be possible to track at least three differently tagged populations without complex experimental controls (e.g., cyan, yellow, and red fluorescent proteins).

We initially performed our experiments with Pantoea eucalypti, a fast-growing strain that does not form biofilms in shaken liquid culture. This allowed us to compare optical density and fluorescence intensity under defined conditions. The growth curves of optical density and fluorescence of bacterial cultures do not resemble each other perfectly, which is not surprising since the rate and maturation of fluorescent proteins is a combination of time-consuming steps from DNA transcription to mRNA translation and protein maturation before the fluorescence signal can be detected (15). This process is, however, reproducible under controlled growth conditions. Furthermore, others have shown that by using a standard curve, it is possible to predict CFU from fluorescence data (13). Our experiments have shown that the constitutive expression of fluorescent proteins is suitable to determine bacterial growth in shaking liquid cultures. Every measure that we tested correlated well with bacterial growth as measured by optical density and cell counts (Tables 1 and 2).

Since growth assessed using fluorescence yields more consistent data than optical density in 96-well plates, it may also be useful in applications like normalization of the fluorescence intensity of bacterial bioreporters (16). Using our workflow, it is possible to conveniently determine the relative fitness of bacteria. Previously, the relative fitness of bacteria has been assessed using shared media reservoirs and filters to separate bacterial populations, which allowed low-throughput investigations of bacterial densities after the competition measured by optical density (17), or by determining the CFU at different sampling times in shared media (e.g., see references 18 and 19). Both methods have in common that they cannot follow population dynamics at high resolution, such as early or late success or bacterial growth rate in the presence of a competing species. By using continuous tracking of bacterial cultures in combination with control experiments that allow normalization, we can infer changes to growth during all phases of an experiment.

To overcome evaporation of the medium, which is one of the major drawbacks of 96-well plates, since it leads to strong edge effects and impacts on optical density, we used hydrophobic, gas-permeable foils to seal 96-well plates. The foils reduced evaporation to negligible amounts over the course of our experiments (up to 2 weeks; data not shown). At the same time, they also led to reduced oxygen availability for bacterial growth and limited the ability to measure optical density due to turbidity of the foil and condensation of droplets on the foil. However, since the reduced oxygen availability impacted the whole plate equally, competition treatments and the respective controls were both similarly impacted, and the resulting relative fitness could still be assessed.

The experimental system is not without flaws, and even though many bacterial strains exhibit negligible autofluorescence in liquid media, some exhibit strong autofluorescence, such as pseudomonads in minimal media (20). To be able to accurately assess the growth of strongly autofluorescent bacteria, it is necessary to determine the autofluorescence of the respective parental strain without the fluorescent protein tag. One clear disadvantage of fluorescent proteins is that many bacterial strains are not amenable to genetic modification. However, several recent studies were able to increase the breadth of bacterial recipients that could be manipulated successfully by using either stable plasmids that do not require antibiotic pressure for medium-term maintenance or (transposon-mediated) chromosomal integration (14, 21, 22). Our system should still be interesting for those hard-to-modify bacteria by competing them against known, fluorescently tagged organisms to determine the impact of the nontagged organism on the tagged one.

### Conclusion.

We have designed a convenient experimental platform that tracks bacterial growth by employing constitutively expressed fluorescent proteins. Our platform allows us to track several fluorescence signals simultaneously in a time-resolved manner and in high replications. It yields more robust results than classical turbidity measurements and can be used to determine competition under controlled conditions.

## MATERIALS AND METHODS

### Strains and growth conditions.

The strains used in this study are listed in Table 3. Bacteria were routinely grown on nutrient broth (NB; HiMedia) or nutrient agar (NA; HiMedia) supplemented with 15 mg liter^−1^ gentamicin, 15 mg liter^−1^ tetracycline, or 10 mg liter^−1^ chloramphenicol where appropriate. Broth cultures were incubated at 30°C in a rotary shaker at 200 rpm. Agar plates were incubated at 30°C. Plasmid pMRE-Tn5-143 was used to transform *Sphingomonas* sp. Fr1 by conjugation, as described previously (14, 23), for constitutive expression of sYFP2 (24). Using the same method, plasmid pMRE-Tn5-145 was used to deliver constitutively expressed fluorescent protein carrying transposons to *Sphingomonas* sp. Fr1, Sphingomonas phyllosphaerae FA2, and *Methylobacterium* sp. strain Leaf85 for constitutive expression of mScarlet-I (25). *Arthrobacter* sp. strain Leaf145 was transformed by sonoelectroporation (26) using the plasmid pKGT-GFP, kindly gifted by Christine Smart (27), for constitutive expression of enhanced GFP (eGFP) (28). *Methylobacterium* sp. Leaf92::Tn*5*-mre145, Methylobacterium radiotolerans 0-1::Tn*5*-mre145, and Pantoea eucalypti 299R::Tn*7*-mre145 were constructed elsewhere (14).

Minimal medium (MM) (1.62 g liter^−1^ NH_4_Cl, 0.2 g liter^−1^ MgSO_4_, 1.59 g liter^−1^ K_2_HPO_4_, 1.8 g liter^−1^ NaH_2_PO_4_·2H_2_O, 15 g liter^−1^ agar, with the following trace elements: 15 mg liter^−1^ Na_2_EDTA_2_·H_2_O, 4.5 mg liter^−1^ ZnSO_4_·7H_2_O, 3 mg liter^−1^ CoCl_2_·6H_2_O, 0.6 mg liter^−1^ MnCl_2_, 1 mg liter^−1^ H_3_BO_3_, 3.0 mg liter^−1^ CaCl_2_, 0.4 mg liter^−1^ Na_2_MoO_4_·2H_2_O, 3 mg liter^−1^ FeSO_4_·7H_2_O, and 0.3 mg liter^−1^ CuSO_4_·5H_2_O) (29) was supplemented with a carbon source at 0.2% (wt/vol). The carbon sources used were sucrose, d-glucose, d-fructose, galactose, d-ribose, xylose, l-arabinose, d-mannitol, sorbitol, glycerol, pyruvate, l-malate, citrate, 2-oxoglutarate, succinate, maleate, fumarate, l-threonine, l-glutamine, l-glutamate, l-serine, l-aspartate, l-proline, l-lysine, l-isoleucine, l-alanine, l-valine, l-tryptophan, l-asparagine, gamma-aminobutyric acid (GABA), and methanol.

### Plate reader experiments.

High-throughput growth experiments were carried out in 96-well microtiter plates (Costar). The 96-well plates were sealed either with a lid or with a hydrophobic gas-permeable membrane (4ti-0516/96; Brooks Life Sciences, Wotton, UK) (gas permeability of 0.6 m^3^ m^−2 ^day^−1^ and water loss of 1 g m^−2 ^day^−1^). For every experiment in microtiter plates, unless stated otherwise, overnight bacterial cultures were used to seed 96-well microtiter plates to reach a volume of 200 μl per well. To that end, cultures were harvested by centrifugation at 6,000 × *g* and washed twice in 1× phosphate-buffered saline (PBS) (8 g liter^−1^ NaCl, 0.24 g liter^−1^ KCl, 1.42 g liter^−1^ Na_2_HPO_4_, 0.24 g liter^−1^ KH_2_PO_4_). Finally, the washed cultures were resuspended and diluted to a defined adjusted optical density at 600 nm (OD_600_). Plates were incubated in a FLUOstar Omega plate reader (BMG Labtech) at 30°C for up to 5 days, depending on the growth rate of each strain and the growth medium. The plates were shaken in meander corner well shaking mode at 300 rpm between each read. Readings of OD_600_ and fluorescence were measured in bottom optic mode. Fluorescence was measured in 2-mm-diameter circles, and the average of eight measurements per well was recorded. Optical densities and the individual fluorescence spectra were measured sequentially to minimize cross talk between the fluorescent proteins. mScarlet fluorescence was excited at 567 to 587 nm, and emission was measured at 613 to 626 nm. sYFP2 and eGFP fluorescence was excited at 475 to 492 nm, and emissions were measured at 511 to 550 nm.

### Growth experiments.

To establish a relationship between fluorescence and optical density, Pe::red was grown in different concentrations of NB in 96-well plates. To that end, a washed culture of Pe::red was resuspended and diluted to an OD_600_ of 0.05. To each well, an aliquot of 20 μl of resuspended culture was added to 180 μl NB or NB diluted to 90, 80, 70, 60, 50, 25, 12.5, 6.75, 3.4, 1.7, or 0% strength. The plate was closed with a lid. The optical density at 600 nm and red fluorescence were measured every 15 min as described above.

Additionally, cultures of the fluorescently tagged strains listed in Table 1 were used to seed microtiter plates containing MM supplemented with individual carbon sources at 0.2% (wt/vol). To that end, every washed culture was resuspended and diluted to an OD_600_ of 0.05, and 20 μl of the resuspended culture was seeded into each well in triplicates for each growth medium. Red or green fluorescence was measured every 15 min, and the OD_600_ was measured at the end of the experiment (final OD_600_).

### Competition experiments and fitness assessment.

Overnight cultures were resuspended to an OD_600_ of 0.37. Four-microliter amounts of the diluted cultures per well were inoculated into 192 μl NB or MM supplemented with 0.2% (wt/vol) succinate in triplicates to perform the competition experiments. Then, the following bacterial mixes were prepared: Pe::red plus 4 μl medium (Pe::red monoculture), Pe::red plus PeWT (nearly isogenic P. eucalypti coculture), Sp::yellow plus 4 μl medium (Sp::yellow monoculture), Sp::yellow plus SpWT (nearly isogenic *Sphingomonas* sp. Fr1 coculture), and Pe::red plus Sp::yellow. In all cases, 96-well plates were sealed with a hydrophobic gas-permeable membrane, and the OD_600_ and red and yellow fluorescence were measured every 15 min.

A similar setup was used to identify potential fitness costs of Tn*7* insertions in Pe::red. To that end, four independent Tn*7* mutant strains were used to compete against a nearly isogenic strain (PeWT) or Sp::yellow.

### Conical-flask experiment.

Two independent Pe::red cultures were grown from single colonies overnight in NB with gentamicin. The overnight cultures were inoculated into 250-ml conical flasks containing NB and gentamicin, resulting in 80 ml of culture at an initial density of an OD_600_ of 0.05. At 0, 0.75, 1.50, 2.50, 3.25, 4, and 24 h postinoculation (hpi), samples of each culture were taken for optical density and red fluorescence measurements, colony counts, and microscopy.

First, 200 μl of culture was transferred into a 96-well plate containing the appropriate blanks. Endpoint measurements were taken for optical density and red fluorescence in triplicates.

Second, 10-fold serial dilutions with dilution factors ranging from 10^2^ to 10^7^ were prepared in triplicate, and 5 μl of each dilution was plated on NB containing gentamicin. Plates were incubated at 30°C overnight and counted under a stereomicroscope.

Finally, an aliquot of each culture was fixed for microscopy. To that end, 1 ml of each of the two cultures was harvested at 15,000 × *g*, washed in 1 ml PBS, and resuspended in 50 μl 1 × PBS. Then, 150 μl paraformaldehyde solution (PFA; 4% [wt/vol]) was added. After 30 min, cells were harvested by centrifugation and resuspended in 50 μl 1× PBS, to which 50 μl 100% (vol/vol) ethanol was added after the cells were resuspended. Fixed samples were stored at −20°C until processed.

### Microscopy.

Slides were prepared by applying 4 μl of each fixed sample onto 0.1% (wt/vol) gelatin-coated microscope slides. Samples were air dried and mounted using 60% glycerol.

Fluorescence microscopy was carried out using a Zeiss AxioImager.M1 fluorescent wide-field microscope at 1,000× magnification and a phase-contrast objective (EC Plan-Neofluar 100×/1.30 Ph3 oil M27 objective) equipped with Zeiss filter set 43HE (BP 550/25-FT 570-BP 605/70), an Axiocam 506, and Zeiss Zen 2.3 software. Images from the first four sampling time points were taken using an exposure of 400 ms, while images of samples at 4 and 24 hpi were taken using exposures of 350 ms and/or 400 ms, respectively. From each independent culture and time point, at least five images containing a total of more than 200 individual cells were taken for image analysis.

Images were processed using ImageJ/FIJI (30). Values were normalized against the exposure time. A mask was created using the IsoData threshold method on the phase-contrast channel and applied to the fluorescence channel; when needed, the mask was adjusted to eliminate any frameshift. The binary process “Open” was applied. Particles were analyzed with the size parameter 0.80 to 2.00 μm^2^, excluding particles touching the border. The average fluorescence intensity of each cell was determined and corrected by subtracting the average background intensity of the respective image. The background intensity of each image was assessed by inverting the previous mask and measuring the median intensity of the image. An average of 678 cells was measured for each sample, with the lowest number of measured cells being attributed to the second biological replicate at 0 hpi (205 cells).

### Data analysis.

Data analysis and visualization were performed using the package *tidyverse* from the R software environment (31, 32). The areas under the curve (AUCs) of the optical density and fluorescence were determined using the function “auc” from the integrated R package *MESS*. Fitness effects in competition experiments were determined by the ratio between the AUC under a certain condition and the mean AUC of a strain growing as a monoculture. Where appropriate, data were fitted into robust simple linear regression models using the function “lmrob” from the R package *robustbase* (33). Pearson’s correlations were determined using the function “cor” from the R package *stats*. Generalized linear mixed models (GLMM) were performed with the R package *gamlss* (34). One- and two-way analyses of variance (ANOVAs) were performed using the function “aov” from the R package *stats*. Post hoc group comparisons were made with the R package *emmeans* (35).
